# Nutritional Factors during and after Cancer: Impacts on Survival and Quality of Life

**DOI:** 10.3390/nu14142958

**Published:** 2022-07-19

**Authors:** Sébastien Salas, Vanessa Cottet, Laure Dossus, Philippine Fassier, Julie Ginhac, Paule Latino-Martel, Isabelle Romieu, Stéphane Schneider, Bernard Srour, Marina Touillaud, Mathilde Touvier, Raphaëlle Ancellin

**Affiliations:** 1 AP-HM, Timone Hospital, Aix Marseille University, 13000 Marseille, France; sebastien.salas@ap-hm.fr; 2 The French Network for Nutrition and Cancer Research (NACRe Network), 78350 Jouy-en-Josas, France; dossusl@iarc.fr (L.D.); p.fassier@eren.smbh.univ-paris13.fr (P.F.); julie.ginhac@inrae.fr (J.G.); paule.latino-martel@inrae.fr (P.L.-M.); iromieu@gmail.com (I.R.); schneider.s@chu-nice.fr (S.S.); b.srour@eren.smbh.univ-paris13.fr (B.S.); marina.touillaud@lyon.unicancer.fr (M.T.); m.touvier@eren.smbh.univ-paris13.fr (M.T.); 3 INSERM UMR1231/CIC 1432, University Hospital, LabEx LipSTIC ANR-11-LABX-0021, University of Burgundy-Franche-Comté, 21000 Dijon, France; 4 International Agency for Research on Cancer, 69000 Lyon, France; 5 Gustave Roussy Institute, 94800 Villejuif, France; 6 Nutritional Epidemiology Research Team (EREN), Sorbonne Paris Nord University, INSERM, INRAE, CNAM, Epidemiology and Statistics Research Centre (CRESS), University of Paris, 93022 Bobigny, France; 7 University Hospital, University of Côte d’Azur, 06000 Nice, France; 8 Léon-Bérard Cancer Centre, UA8 Inserm, 69000 Lyon, France; 9 The French National Cancer Institute (INCa), 92012 Boulogne-Billancourt, France; rancellin@institutcancer.fr

**Keywords:** cancer, diet, dietary supplements, obesity, alcohol

## Abstract

The French National Cancer Institute conducted a collective expertise study with researchers and clinical experts from the French Network for Nutrition And Cancer Research (NACRe Network). The objective was to update the state of knowledge on the impacts of nutritional factors on clinical endpoints during or after cancer. Data from 150 meta-analyses, pooled analyses or intervention trials and 93 cohort studies were examined; they concerned 8 nutritional factors, 6 clinical events and 20 cancer locations. This report shows that some nutritional factors have impacts on mortality and on the risks of recurrence or second primary cancer in cancer patients. Therefore, high-risk nutritional conditions can be encountered for certain cancer sites: from the diagnosis and throughout the health care pathways, weight loss (lung and esophageal cancers), malnutrition (lung, esophageal, colorectal, pancreatic, gastric and liver cancers), weight gain (colorectal, breast and kidney cancers) and alcohol consumption (upper aerodigestive cancers) should be monitored; and after cancer treatments, excess weight should be detected (colorectal, breast and kidney cancers). These situations require nutritional assessments, and even support or management by health care professionals, in the context of tertiary prevention. This report also highlights some limitations regarding the existing literature and some needs for future research.

## 1. Introduction

In the United States, an estimated 16.9 million individuals with a history of cancer were alive in 2019, and it is estimated that in France, 3.8 million people are living with or have recovered from cancer [1]. Advances in screening, diagnosis and treatment have improved patient survival for most cancers [2]. The goal of cancer management is no longer just to treat the disease but also to reduce the risks of further morbidity and mortality. To pursue this objective, the French National Cancer Institute, a health and science agency dedicated to cancer and placed under the umbrella of the Ministries of Health and Research, called for a generalized preventive approach after a cancer diagnosis, including in particular smoking cessation, the promotion of appropriate nutritional behaviour and the reduction of alcohol consumption. A cancer diagnosis appears to be an opportune time to adopt healthier behaviours based on the particular reception of risk reduction messages by patients [3,4].

Nutritional factors (weight, diet, physical activity, alcohol) are identified as impacting the onset of cancer [5]. They are also involved in prognosis, quality of life, co-morbidities, recurrences and second cancers, although the literature is more recent and less abundant. Based on a review of the scientific literature, in 2012, the American Cancer Society published nutrition and physical activity guidelines for cancer patients that highlight the benefits of a diet rich in plant products and whole grains, regular physical activity and maintaining a healthy weight [6].

Recently, the French National Institute of Cancer (INCa) conducted reviews of the scientific literature on the benefits of physical activity and the benefits of smoking cessation in cancer patients to inform and educate health professionals and patients [2,7]. In line with this approach, INCa initiated a collective expert evaluation to update the American Cancer Society’s 2012 analysis to complete the information provided to physicians and patients on the influence of nutritional factors during and after cancer.

## 2. Materials and Methods

### 2.1. Evaluation Process

An expert group was set up at the end of 2017 by INCa involving clinicians and epidemiologists from the French network on Nutrition And Cancer Research (NACRe network, https://www6.inrae.fr/nacre/Accueil/NACRe-network, accessed on 15 June 2022), chosen for their expertise in the field. The modalities for the systematic literature review and the evaluation of the evidence were discussed and adopted by the expert group. The nutritional factors and clinical events to be considered were defined, and the types of studies to include in the review and the selection criteria for publications were also specified. The bibliographic queries/search strategies were then developed according to these criteria. 

The method of bibliography analysis, the nature of data to be extracted from articles, the analysis grids and the criteria for evaluating the levels of evidence for the relationships between nutritional factors and clinical events were also discussed by the expert group.

### 2.2. Definitions and Criteria for Inclusion/Exclusion of Publications

The inclusion criteria were:

- Population: cancer survivor or patient under neoplastic treatment.
- Nutritional factors of interest: weight (loss or gain)/body mass index/cachexia/body composition (fat mass, muscle mass, body surface area), dietary patterns (a posteriori and a priori), alcoholic beverages, dietary supplements (vitamins, minerals and other nutrients or plants), foods, medicinal plant or mushroom products, nutritional advice and nutritional advice combined with physical activity.
- Type of study: intervention trials, meta-analyses, pooled analyses published until February 2019. For nutritional factors and cancer sites with insufficient data from meta-analyses, pooled analyses and intervention trials (Request A in Appendix A) cohort studies with more than 300 subjects published until November 2018 (Request B in Appendix A) were included. This was the case for:
  - Weight and associated factors: endometrium, cervix, liver, pancreas;
  - Weight, undernutrition and associated factors: head and neck, pharynx, nasopharynx;
  - Dietary patterns: all cancer sites;
  - Alcoholic beverages: all cancer sites except breast;
  - Foods: all cancer sites (except soya, fruit and vegetables and fibre for breast cancer only);
  - Vitamins and minerals (except vitamin C and antioxidants for breast cancer only) or herbs: all cancer sites;
  - Timing of exposure: at or after diagnosis;
  - Clinical outcomes: overall mortality, cancer-specific mortality, second primary cancer, cancer progression, cancer recurrence, quality of life.

Exclusion criteria were:

- Precancerous lesions as outcomes: colonic adenomas, polyps, polycystic ovary syndrome, cervical dysplasia (Cervical Intraepithelial Neoplasia Grade 1 or CIN1);
- Primary prevention studies;
- Animal or in vitro studies;
- Studies in children;
- Studies with nutritional exposure measured before diagnosis; for meta-analyses, publications not distinguishing pre- and post-diagnosis exposures.
- Nutritional factors excluded: therapeutic food modes, chewing gum, water, non-validated criteria for the diagnosis of undernutrition (Inflammatory Nutrition Index, Nutritional Index, Subjective Global Assessment, albumin...); this expertise did not include the impacts of medical nutrition therapy such as enteral/parenteral nutrition, immuno-nutrition, peri-operative nutrition or oral nutritional supplements. In this field, recommendations for the nutritional management of adult cancer patients have been developed [8]. As physical activity during and after cancer has already been the subject of a recent collective expertise report [7], this factor was not included in this work, and only interventions combining nutritional advice and physical activity were considered.
- Clinical outcomes/events excluded: biomarkers (inflammation, albumin, immune function, Prostate-specific antigen, oxidative stress, etc.), side or intermediate effects (diarrhoea, mucositis, intestinal function, arthralgia, neuropathies, pain, weight loss, loss of muscle mass, etc.), pathologic complete response, short-term mortality, biological recurrence, quality of life criteria other than overall quality of life.
- Absence of hazard ratio, relative risk or odds ratio and of their 95% confidence interval.
- Non-randomized intervention trials.

### 2.3. Bibliographic Queries, Data Extraction and Analyses

The literature search was conducted in the PubMed database between August 2010, the date of the bibliographic search of the American Cancer Society Review [6], and February 2019 (Nov. 2018 for cohort). It used MeSH terms for indexed articles and free words in the title and abstract fields, restricted to English or French. Keywords and queries are provided in Appendix A. In addition, meta-analyses of the World Cancer Research Fund/American Institute for Cancer Research 2014 report on the relationships between nutrition and survival in breast cancer patients were used. Reviews were also used for completeness of the bibliography. To avoid redundancy, if there were several meta-analyses for the same exposure and cancer site, taking into account the same studies, only the most recent or highest-quality meta-analysis was considered. Similarly, if studies (intervention trial or cohort study) were already included in a retained meta-analysis, they were not selected.

The articles to be analysed were distributed among experts according to their respective competences, and any uncertainties were resolved by discussion within the expert group. For each nutritional factor considered, the expert first reviewed available publications based on title, abstract and keywords and then on the full text of the article. For the meta-analyses and pooled analyses, a double reading was carried out by two experts. Using a standardized data collection form, each expert extracted the following information from the full-text selected articles: for all studies, first author’s last name, publication year, country, type of study, inclusion/exclusion criteria, sample size, mean age, gender, cancer site, number of cancer cases, mean follow-up, exposure, outcome, groups’ comparison or dose–response relationship and corresponding hazard ratios or relative risks, and 95% confidence intervals, adjustment factors and bias; for meta-analyses or pooled analyses: number and type of studies included, stratification, heterogeneity for meta-analyses and pooled analyses, sensitivity analyses and results from meta-regressions; for intervention trials: name of the trial, country in which the trial was conducted, randomization, blindness, intervention (type, duration), follow-up duration and number/proportion of subjects lost to follow-up.

The summary of extracted data and the updated level of evidence proposed by each expert were reviewed and discussed by the overall expert group until a consensus was reached. Finally, the levels of evidence were qualified as convincing, probable, suggestive or not conclusive, according to the criteria that were defined in Table 1. The expert group corrected the drafts of each expert and validated the report. A national consultation was carried out with 103 external independent experts with the objective of assessing the readability, coherence and acceptability of the text, conclusions and recommendations.

### 2.4. Terminology and Definitions

In the report, the terms below have been used with the following definitions: 

Dietary supplements: According to European Directive 2002/46/EC, food supplements are “foodstuffs intended to supplement the normal diet and which constitute a concentrated source of nutrients or other substances with a nutritional or physiological effect, alone or in combination, marketed in dose form, namely, forms of presentation such as capsules, pastilles, tablets, pills and other similar forms, as well as sachets of powder, ampoules of liquid, dropper bottles and other similar forms of liquid or powder preparations intended to be taken in measured units of small quantities”. They consist of one or more compounds that may include nutrients such as vitamins, minerals, amino and fatty acids and plant extracts.

Body composition: the following parameters have been found in studies of body composition:

- Body Surface Area: the external surface area of the skin covering the body (m²). The most common calculation formula is the square root of (weight x height/3600), with weight in kg, height in cm and Body Surface Area in m². The standard is 1.73 m².
- Skeletal Muscle Index: muscle mass index (in cm²/m²), calculated as the ratio of skeletal muscle area (cm²) to the square of body size (m²) or the square of body surface area (m²) depending on the indices used. The cross-sectional skeletal muscle area is measured on a CT scan cross-section at the third lumbar vertebra and is a reliable representation of the total muscle mass of the body.
- Skeletal Muscle radioDensity: muscle radiodensity, the average of the attenuation coefficient in the muscle (expressed in Hounsfield unit, which is measured on a CT scan cross-section at the third lumbar vertebra. The attenuation of the muscle is expressed in relation to that of the water taken as a reference).

## 3. Results

**Figure 1:**
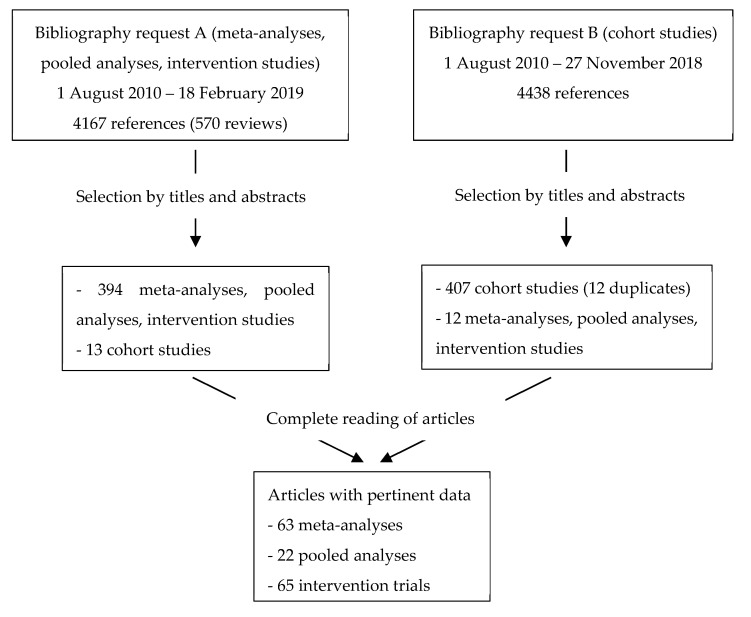
Flow chart.

A total of 8605 references were identified by searches in the Medline database, from which 826 abstracts were selected (Figure 1). After reading the corresponding full- text articles, 243 relevant articles were identified, including 63 meta-analyses, 22 pooled analyses, 65 intervention trials and 93 cohort studies.

The full results and analysis of this literature search are detailed in the INCa report published by the expert group. In particular, the synthetic tables present all studies and their main results by nutritional factor and cancer location. In the following sections, the indicators of each nutritional factor, the results and the conclusions of the evaluation process by the expert group are summarized for cancers sites for which a *convincing*, a *probable* or a *suggested* level of evidence was established. We also highlighted main nutritional factors with only limited evidence, in order to foster and guide further research in the field. Table 2 is a synthetic table that summarizes the level of evidence for the associations between each nutritional factor and the studied clinical events during and after cancer for each cancer site. For a large majority of the studied relationships, the levels of evidence are judged “inconclusive” and require further research to consolidate knowledge about these associations.

### 3.1. Overweight and Obesity

Overweight or obesity after cancer diagnosis may be either beneficial or deleterious depending on the cancer location.

Obesity in women with non-metastatic breast cancer was associated with an increased risk of second cancer (*convincing* level of evidence) and increased overall and cancer-specific mortality (*probable* level of evidence) [9,10,11,12,13]. Overweight and obesity in these patients were probably associated with an increased risk of recurrence [10]. Obesity (but not overweight) in patients with colorectal cancer was *convincingly* associated with an increased overall mortality and risk of relapse [14,15,16,17]. Overweight and obesity were associated with increased overall mortality in patients with kidney cancer (*probable* level of evidence) [18].

In contrast, both overweight and obesity were associated with a reduced overall and cancer-specific mortality in lung cancer patients, as well as with a reduced overall mortality in patients with esophageal cancer (*probable* level of evidence) [19,20]. One study *suggested* an association between overweight and obesity and a decreased cancer-specific mortality in stomach cancer patients [21]. Other limited data *suggested* an association between weight gain and reduced overall mortality or risk of progression in lung cancer patients [22].

### 3.2. Malnutrition and Sarcopenia

Malnutrition at diagnosis can translate into a low body mass index, weight loss or sarcopenia (i.e., myopenia in the concerned studies). Malnutrition was *convincingly* associated with an increased overall mortality, risk of relapse and progression in patients with colorectal cancer [15,17,23,24].

Malnutrition was also associated with an increased overall mortality in patients with lung and stomach cancer, and with esophagus, liver and pancreas cancer when malnutrition was evaluated by sarcopenia. Malnutrition evaluated by sarcopenia was also associated with an increased cancer-specific mortality in stomach cancer and with the risk of relapse in liver and stomach cancer patients (*probable* level of evidence) [20,25,26,27,28,29].

Other limited data *suggested* that malnutrition was associated with an increased overall mortality in cervical and kidney cancer patients, an increased cancer-specific mortality in lung cancer patients, an increased risk of relapse in patients with upper aerodigestive tract cancers, an increased overall mortality and increased risk of relapse in nasopharyngeal cancer patients, an increased overall and cancer-specific mortality and increased risk of relapse in patients with solid tumors, and an increased global mortality and increased risk of progression in patients with hematological tumors [20,30,31,32,33,34,35,36,37].

Limited evidence suggested that nutritional advice to limit weight loss was associated with decreased cancer-specific mortality and risk of relapse in colorectal cancer patients (*suggested* level of evidence) [38].

### 3.3. Alcoholic Beverages

Few studies have evaluated the relationships between alcohol consumption following diagnosis of cancer and cancer prognosis. The level of evidence was judged as *probable* for the association between alcohol and the risk of a second cancer in patients with cancer of the upper aerodigestive tract [39].

### 3.4. Dietary Patterns

Since foods are not eaten in isolation, considering the effects of dietary patterns allows for assessing the overall role of diet as a combination of different foods and nutrients. 

Some dietary profiles and foods may be beneficial for certain cancer sites. A low-fat diet was associated with decreased overall mortality and risk of relapse in breast cancer patients (*probable* level of evidence) [40,41]. Data regarding the Mediterranean diet (a priori score or a posteriori calculation) were too scarce and inconsistent. In some studies, a score of adherence to specific nutritional recommendations (ACS: American Cancer Society; WCRF: World Cancer Research Fund; HEI: Healthy Eating Index; DASH: Dietary Approaches to Stop Hypertension…) was used to explore the effect of diet on prognostic of patients in addition to other health behaviors (physical activity, alcohol consumption…), but the level of evidence was not sufficient to conclude so far.

### 3.5. Foods

For prostate cancer patients, limited data *suggested* that saturated fatty acids were associated with increased overall mortality and high-fat dairy products with an increased cancer-specific mortality, while vegetable fats were associated with decreased overall mortality (*suggested* level of evidence) [42,43,44,45]. Soy consumption following a diagnosis of breast cancer was associated with a decreased risk of recurrence (*suggested* level of evidence) [46,47,48]. The consumption of fiber-containing foods following a diagnosis of breast cancer was associated with decreased overall mortality (*probable* level of evidence) [49]. Limited evidence *suggested* that coffee consumption following a diagnosis of colorectal cancer was associated with decreased overall mortality [50,51].

### 3.6. Dietary Supplements

Some dietary supplements taken under medical supervision after a diagnosis of cancer may be beneficial for some cancer sites. In breast cancer patients, the consumption of vitamin C supplements was associated with decreased overall and cancer specific mortality (*probable* level of evidence) [52]. Limited evidence suggested that the consumption of vitamin D or E supplements was associated with a decreased risk of breast cancer recurrence (*suggested* level of evidence) [53]. The consumption of branched-chain amino acid supplements was associated with decreased overall mortality in patients with liver cancer (*suggested* level of evidence), but this factor should be considered with caution regarding the possible metabolic or vascular risk evocated in the literature [54,55]. In contrast, deleterious effects have been highlighted by the American Cancer Society associated with the consumption of vitamin E-based dietary supplements in patients with upper aerodigestive tract cancers (increased risk of overall and specific mortality) [6]. Moreover, high doses of antioxidants repaired the oxidative damage induced by cancer cell treatments, thus limiting the effectiveness of these treatments [48,56,57,58].

### 3.7. Medicinal Plants and Chinese Mushrooms

Limited data exist on the impacts of certain Chinese mushrooms and medicinal plants after a diagnosis of cancer. Researchers have suggested that extracts of Coriolus versicolor fungi are associated with a decrease in overall mortality in patients with breast, colorectal and stomach cancer (*suggested* level of evidence) [59]. Chinese herbal decoctions of Jianpi Qushi and Jianpi Jiedu were associated with improved overall quality of life in patients with colorectal cancer (*suggested* level of evidence) [60,61]. However, these results have only been observed in meta-analyses of small trials including Asian patients exclusively and should therefore be interpreted with caution.

## 4. Discussion

Measuring the impacts of nutritional factors in cancer patients on the prognosis or progression of the disease is complex due to the diversity of situations that may be encountered depending on the disease (stage, site or type of tumors), the treatments administered or the associated side effects, which may interact with nutritional factors. It is thus difficult to conclude with certainty, in the case of observational studies, that an observed association is not due to a possible unidentified confounding factor or to reverse causality (the nutritional factor is affected by the disease and not the other way around). The majority of studies are based on volunteer participants, described in the literature as not being representative of the cancer population because they are usually “in better shape” and already aware of or motivated by the issues of the study. This leads to a potential selection bias that limits the extrapolation of these results to the entire cancer population. In weight-related studies, the inherent limitations are, on the one hand, the difficulty in distinguishing between intentional and unintentional (disease-related) weight loss, particularly in breast cancer patients, and, on the other hand, the difficulty in considering the problem and the lack of studies on muscle loss in obese subjects (sarcopenic obesity). More generally in the case of deleterious factors (undernutrition, alcohol...), it is not possible for ethical reasons to conduct intervention trials exposing patients to these factors. It may be considered (if the expected benefit is significant) to make interventions to reduce exposure to risk factors, for example by giving nutritional advice to limit weight loss. No intervention studies assessed the combined effects of nutritional advice and physical activity on survival, recurrence or secondary cancer. It is important to complete the available data. This information would be essential for defining programs well adapted to patients’ profiles. The type of intervention (contents of nutritional advice, frequencies and modalities —phone exchange, meeting, therapeutic education program …) would also have to be specified. Other methodological limitations of the studies identified in this review are common, such as: limited sample size; duration of follow-up often too short to conclude on the effects of the intervention, especially for cancer sites with good prognosis; unspecified or uncertain timing of exposure measurements (before/after diagnosis, before/after treatment/after treatment), which are nonetheless crucial to consider; elderly populations not individualized in the studies; different reference thresholds used for the same exposure factor; the possible lack of adjustment for tobacco and/or alcohol in some studies on the role of weight in patients with lung cancer; and the lack of control for other prognostic factors for the disease and the heterogeneity of the populations studied (e.g., in breast cancer, very different prognosis according to molecular type, not taking into account menopausal status).

This shows the extent of current research needs. First of all, the temporality of exposure in relation to cancer diagnosis is important to consider. Additional studies are needed regarding post-diagnosis exposure periods in order to make recommendations on the expected benefits/risks of changes in nutritional behavior following a cancer diagnosis. In particular, it will be essential to be able to provide answers to the following questions: What is the effect of stopping alcohol consumption after cancer diagnosis? What is the effect of intentional weight loss after diagnosis?

Most studies have been conducted in breast cancer patients. It is therefore necessary to be able to provide answers for other cancer sites or for different types and stages of the disease. For certain factors (certain dietary supplements, mushrooms, Chinese medicinal plants), the studies were conducted exclusively in Asian populations. They should therefore be confirmed in European/other populations and in the conditions of therapeutic management that are common in Europe. The biological mechanisms by which nutritional factors could prevent cancer recurrence or mortality are still uncertain. In the future, the identified mechanisms may be taken into account in the evaluation of the levels of evidence. 

In setting levels of evidence and developing recommendations, the working group has taken these different limitations into account. Wherever possible, levels of evidence have been established for associations between the nutritional factors and the clinical events of interest for each cancer site. When studies included patients with different cancers and provided results across all cancers, the consistency of the results with those by cancer site was verified. However, given the heterogeneity of these different clinical situations, the results for all cancers combined did not raise the levels of evidence. Similarly, for weight-related studies, when a mixed group including both overweight and underweight patients was used as a control group, levels of evidence were not evaluated. 

The potential deleterious effects of certain factors, in particular interactions with treatments that may reduce their effectiveness, have already been the subject of recommendations for caution, such as: high-dose antioxidant intake [6], alcohol consumption [6] and consumption of soy in the form of food or dietary supplements [47,48]. The working group convened by INCa also raised concerns about the potential risks and regulatory aspects related to Chinese medicinal mushrooms and plants, as they are currently not authorized in Europe for human consumption. Despite these limitations, the data collected for certain factors justify certain general nutritional prevention recommendations for patients suffering from or cured of cancer, unless specific medical advice is given. For a large majority of the relationships studied, the established levels of evidence are inconclusive and require further research to consolidate knowledge about these associations.

## 5. Conclusions

These recommendations are based on the experts’ evaluation and on clinical practice. Only convincing and probable levels of evidence allow for recommendations to be proposed. Nutritional management, when necessary, should be adapted to the patient’s clinical situation. The recommendations do not replace the need to assess the patient’s nutritional status throughout the care pathway.

The expert group considered that it is inappropriate during treatment to make obese patients lose weight because of the associated risk of muscle loss and undernutrition. Return to a normal weight can only be considered in patients after treatment. Similarly, in people over 70 years of age, weight loss should be avoided both during and after treatment. Available data for breast and colorectal cancer patients showed the deleterious effects of both obesity and undernutrition. It is important to prevent, detect and, if necessary, manage malnutrition in these patients: during treatment, it is recommended to avoid weight gain in patients who are not malnourished; after treatment, it is recommended to maintain or reach a normal weight. For kidney cancer patients, the available data show the deleterious effects of overweight and obesity: during treatment, it is recommended that weight gain be avoided in patients who are normal or overweight; after treatment, it is recommended to maintain or achieve a normal weight. For patients with cancer of the liver, pancreas and stomach, available data show the deleterious effect of undernutrition. For these patients, after diagnosis, it is recommended to prevent, detect and, if necessary, treat undernutrition. For patients with lung and esophageal cancer, the available data show both a beneficial effect of overweight and a deleterious effect of undernutrition. In these patients, it is recommended to avoid weight loss and to prevent, detect and, if necessary, manage undernutrition.

As in the general population, the data suggested that high-fat foods be limited and that fiber-rich foods be preferred. For patients with upper aerodigestive tract cancer, it is recommended to avoid alcohol consumption. Even in the absence of conclusive evidence for the other locations, it is recommended that all cancer patients limit their alcohol consumption. Although the level of evidence has been described as probable for the consumption of soy and vitamin C supplements, in the absence of specific information on amounts, duration and possible deleterious interactions with treatment, it is premature to recommend their consumption by breast cancer patients.

**Figure 2:**
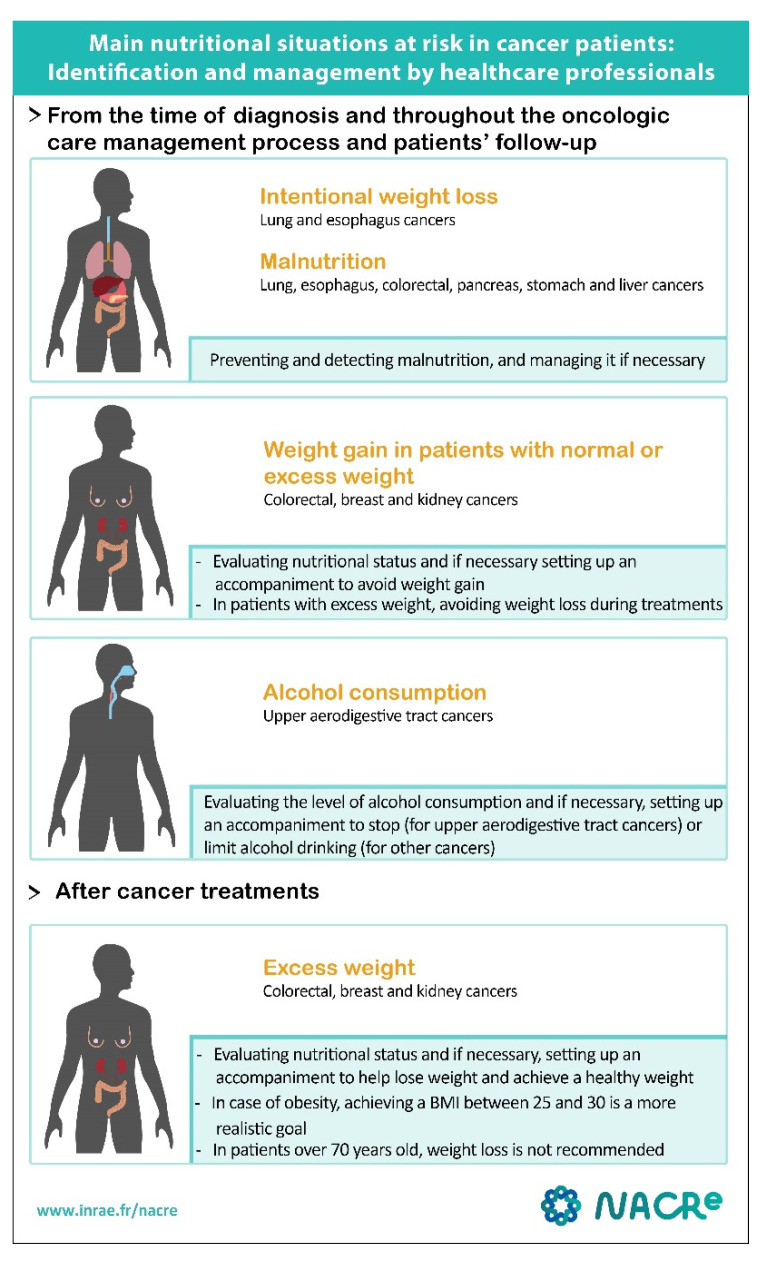
Recommendations: Identification and management of the main nutritional situations at risk in cancer patients by healthcare professionals. Copyright permission has been obtained from the national NACRe Network (Nutrition And Cancer REsearch network).

Regarding the use of Chinese mushrooms and medicinal plants after a cancer diagnosis, the effects on prognosis of cancer should be evaluated in European populations and under the conditions of therapeutic management in Europe, and it should be verified that there are no deleterious interactions with anti-cancer treatments. Currently, extracts of the fungus Coriolus versicolor and the plants Jianpi Qushi and Jianpi Jiedu are not authorized for human consumption in Europe. In the current state of knowledge, and taking into account the reservations mentioned, it is recommended that patients be advised not to self-administer these extracts or decoctions during cancer treatments. In conclusion, the recommendations for the main risky nutritional situations that heath care professionals should manage are summarized in Figure 2.

## Figures and Tables

**Table 1:** Criteria used by the INCa expert for evaluating the levels of evidence.

Grade	Criteria Required
Convincing	MA or PA of intervention studies or at least 2 intervention studies with: - Statistically significant association - Controlled and randomized - High number of patients and events - No high or unexplained heterogeneity * Or MA or PA of prospective cohort studies with: - Statistically significant association - Dose–response analysis - High number of studies included in the MA - High number of patients and events - No high and unexplained heterogeneity
Probable	MA or PA of intervention studies or at least 2 intervention studies with: - Statistically significant association - Controlled and randomized - High number of patients and events Or MA or PA of prospective studies with: - Statistically significant association - High number of studies included - High number of patients and events - No high and unexplained heterogeneity
Suggestive	MA or PA of intervention studies or one intervention with: - Statistically significant association - Controlled and randomized - High number of patients and events Or MA or PA of prospective studies with: - Statistically significant association Or At least 2 cohort studies with: - Statistically significant association - High number of patients and events
Not conclusive	- Not enough studies or - Inconsistent results or - High heterogeneity or - Low number of patients or events or - Poor-quality studies
Improbable	MA or PA of intervention studies or at least 2 intervention studies with: - No statistically significant association: relative risk near 1 and narrow confidence interval - Controlled and randomized - High number of patients and events - No high and unexplained heterogeneity * Or MA or PA of prospective studies with: - No statistically significant association: relative risk near 1 and narrow confidence interval - High number of studies included, - High number of patients and events - No high and unexplained heterogeneity

MA: meta-analysis; PA: pooled analysis; * High heterogeneity: *I*^2^ ≥ 75% (WCRF/AICR, 2007).

**Table 2:** (color). Levels of evidence for the associations between nutritional factors and clinical events during and after cancer for different locations.

		Breast						Colo-rectum					Prostate		Lung			Esophagus	
		M		SPE-M	R	SPC		M	SPE-M	R	P	QoL	M	SPE-M	M	SPE-M	P	M	
Excess body weight	Overweight				1														
	Obesity			2	3			4											
	Overweight + Obesity																		
	Weight gain																		
Malnutrition	Underweight																		
	Weight loss																		
	Body composition																		
	Sarcopenia																		
Alcoholic beverages																			
Foods	Soja				∆														
	Fibers																		
	Coffee																		
	High-fat dairy products																		
	Saturated Fatty Acids																		
	Vegetable fats																		
Dietary Patterns	Low-fat diet																		
Nutritional advices	To limit weight loss																		
Dietary supplements	Vitamin C	∆		∆															
	Vitamin D				∆														
	Vitamin E				∆														
	Branched chain amino acids																		
Medicinal plants and Chinese mushrooms	Coriolus versicolor (extracts)	∆						∆											
	Jianpi Qushi (decoctions)											∆							
	Jianpi Jiedu (decoctions)											∆							
		**Liver**		**Pancreas**		**Stomach**			**Cervix**	**Bladder**	**UADT**		**Nasopharynx**		**Solid tumors**			**Hematological tumors**	
		**M**	**R**	**M**		**M**	**SPE-M**	**R**	**M**	**M**	**R**	**SPC**	**M**	**R**	**M**	**SPE-M**	**R**	**M**	**P**
Excess body weight	Overweight																		
	Obesity																		
	Overweight + Obesity																		
	Weight gain																		
Malnutrition	Underweight																		
	Weight loss																		
	Body composition																		
	Sarcopenia																		
Alcoholic beverages																			
Foods	Soja																		
	Fibers																		
	Coffee																		
	High-fat dairy products																		
	Saturated Fatty Acids																		
	Vegetable fats																		
Dietary Patterns	Low-fat diet																		
Nutritional advices	To limit weight loss																		
Dietary supplements	Vitamin C																		
	Vitamin D																		
	Vitamin E																		
	Branched chain amino acids	∆																	
Medicinal plants and Chinese mushrooms	Coriolus versicolor (extracts)					∆													
	Jianpi Qushi (decoctions)																		
	Jianpi Jiedu (decoctions)																		
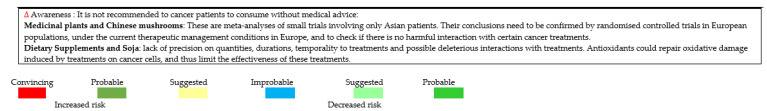																			

M: overall mortality; spe-M: specific mortality; R: cancer recurrence; SPC: second primary cancer, P: progression; QoL: Quality of Life; UADT: upper aerodigestive tract cancers; 1: overweight 4 years after diagnostic, among RE+ patients; 2: obesity (IMC ≥ 35 kg/m^2^); 3: obesity 2 years and et 4 years after diagnostic, among RE+ patients; 4: suggested reduction for metastatic cancers.

## Data Availability

Not applicable.

## Appendix A. Bibliographic Requests

#1 cancer and cancer patients

(“Cancer Survivors”[Mesh] OR (neoplasms[Mesh] OR cancer[tiab] OR cancers[tiab] OR tumor[tiab] OR tumors[tiab] OR tumour[tiab] OR tumours[tiab] OR Neoplas*[TIAB] OR Carcino*[TIAB] OR Sarcom*[TIAB] OR Malign*[TIAB] OR Adenocarcinom*[TIAB] OR Adenosarcom*[TIAB] OR Angiosarcom*[TIAB] OR Gliom*[TIAB] OR Leukaem*[TIAB] OR Leukem*[TIAB] OR Lymphom*[TIAB] OR Melanom*[TIAB] OR myeloma*[tiab] OR glioblastoma*[tiab]) AND (patient[tiab] OR patients[tiab] OR survivor*[tiab] OR “Survivors”[Mesh] OR diagnos*[tiab] OR recurrence[tiab]))

#2 Nutritional factors:

#2.1 Weight

((“Body Weights and Measures”[Mesh] NOT (Organ Size [Mesh] OR Tumor Burden [Mesh])) OR Body Weight Changes[Mesh] OR weight loss[tiab] OR adiposity[tiab] OR body fatness[tiab] OR weight change[tiab] OR weight gain[tiab] OR BMI[tiab] OR “body mass index”[tiab] OR obes*[tiab] OR overweight[tiab] OR over-weight[tiab] OR “waist hip ratio”[tiab] OR anthropometry[tiab] OR underweight[tiab] OR under-weight[tiab] OR WHR[tiab] OR waist circumference[tiab] OR hip circumference[tiab] OR body composition[tiab] OR body mass[tiab] OR skinfold measurement*[tiab] OR skinfold thickness[tiab] OR bio-impedence[tiab] OR bioimpedence[tiab] OR body fat composition[tiab] OR corpulence[tiab])

#2.2 Dietary patterns

(Diet, Mediterranean[Mesh] OR Diet, Western[Mesh] OR Healthy Diet[Mesh] OR Diet, Vegetarian[Mesh] OR ((Nutrition*[TIAB] OR Diet*[TIAB] OR Eating[TIAB] OR Food[TIAB] OR Prudent[TIAB] OR Mediterranean[TIAB] OR Western[TIAB] OR Healthy[TIAB] OR Unhealthy[TIAB]) OR PNNS[TIAB] OR “WCRF/AICR”[Tiab]) AND (Pattern[TIAB] OR Patterns[TIAB] OR Profile[TIAB] OR Profiles[TIAB] OR Score[TIAB] OR Scores[TIAB] OR index[TIAB] OR indexes[TIAB])) OR ((Prudent[TIAB] OR Mediterranean[TIAB] OR Western[TIAB] OR Healthy[TIAB] OR Unhealthy[TIAB]) AND (Diet[TIAB] OR Diets[TIAB])) OR (Healthy eating index[TIAB] OR HEI score[TIAB] OR AHEI[TIAB] OR Diet quality index[TIAB] OR Dietary quality index[TIAB] OR DQI-I[TIAB] OR DQI-R[TIAB] OR Programme National Nutrition Santé-Guideline Score[TIAB] OR PNNS-GS[TIAB] OR “medi-lite”[Tiab] OR Healthy food index[TIAB] OR Mediterranean adequacy index[TIAB] OR Dietary guideline index[TIAB] OR Diet quality score[TIAB] OR MDQI[TIAB] OR aMed score[TIAB] OR MDS score[TIAB] OR rMED score[TIAB] OR MSDPS[TIAB] OR Pandiet[TIAB] OR DASH[TIAB] OR Dietary Approaches to Stop Hypertension[TIAB]))

#2.3 alcoholic beverages

(Alcohol-Related Disorders[Mesh] OR Alcohol Drinking[Mesh] OR Alcoholic Beverages[Mesh] OR Binge Drinking[Mesh] OR Alcoholism[Mesh] OR Alcoholics[Mesh] OR Alcoholic Intoxication[Mesh] OR Temperance[Mesh] OR Ethanol[Mesh] OR Acetaldehyde[Mesh] OR (alcohol[Title/Abstract] OR alcohols[Title/Abstract] OR alcoholic[Title/Abstract] OR alcoholics[Title/Abstract] OR alcoholism[Title/Abstract] OR drunkenness[Title/Abstract] OR ethanol[Title/Abstract] OR acetaldehyde[Title/Abstract] OR temperance[Title/Abstract] OR cider[Title/Abstract] OR wine[tiab] OR beer[tiab] OR spirits[tiab] OR liquor[tiab] OR liquors[Title/Abstract])

#2.4 dietary supplements

Dietary Supplements[Mesh] OR (supplement*[tiab] AND (diet*[tiab] OR nutrition*[tiab] OR vitamin*[tiab] OR multivitamin*[tiab] OR Antioxidants[Mesh] OR Antioxidant*[tiab] OR Antioxidants[Pharmacological Action] OR “Antioxidants”[Substance Name] OR “Vitamins”[Mesh] OR “Vitamins” [Pharmacological Action OR folates[TIAB])

#2.5 Foods

Diet, Food, and Nutrition[MH] OR Food and beverages[MH] OR Dietary Fats[Mesh] OR cereal*[TIAB] OR grain*[TIAB] OR granary[TIAB] OR wholegrain*[TIAB] OR wholewheat[TIAB] OR fiber[TIAB] OR fibre[TIAB] OR vegetable*[TIAB] OR legume*[TIAB] OR fruit*[TIAB] OR citrus[TIAB] OR grapefruit[TIAB] OR tomato*[TIAB] OR beans[TIAB] OR soy[TIAB] OR soybean*[TIAB] OR soyabean*[TIAB] OR soyfood[TIAB] OR soya[TIAB] OR nut[TIAB] OR nuts[TIAB] OR peanut*[TIAB] OR groundnut*[TIAB] OR meat[TIAB] OR poultry[TIAB] OR chicken[TIAB] OR fish[TIAB] OR egg[TIAB] OR eggs[TIAB] OR bread[TIAB] OR fats[TIAB] OR oils[TIAB] OR oil[TIAB] OR seafood[TIAB] OR syrup[TIAB] OR dairy[TIAB] OR milk[TIAB] OR butter[TIAB] OR yogurt[TIAB] OR yoghurt[TIAB] OR cheese[TIAB] OR herbs[TIAB] OR spices[TIAB] OR chilli[TIAB] OR chillis[TIAB] OR pepper*[TIAB] OR condiments[TIAB] OR chocolate[TIAB] OR cacao[TIAB] OR salt[TIAB] OR salting[TIAB] OR salted[TIAB] OR salty[TIAB] OR saltfree[TIAB] OR sodium chloride[TIAB] OR garlic[TIAB] OR beverage*[TIAB] OR water[TIAB] OR drink*[TIAB] OR tea[TIAB] OR coffee[TIAB] OR juice[TIAB] OR foods[TIAB] OR broccoli[TIAB] OR pomegranate[TIAB] OR mushroom[TIAB] OR mushrooms[TIAB] OR cruciferous[TIAB] OR sugar[TIAB] OR meats[TIAB] OR algae[TIAB] OR glycemic load[MH] OR glycemic index[MH] OR glycemic load[TIAB] OR glycemic index[TIAB]

#2.6 Nutritional advices

(“dietary counseling”[tiab] OR “Dietary advice”[tiab] OR “Dietary intervention”[tiab] OR “Nutritional intervention”[tiab] OR “Nutrition intervention”[tiab] OR “Nutrition counseling”[tiab] OR “Nutritional counseling”[tiab] OR “Diet counseling”[tiab] OR Dietitian[tiab] OR Dietitians[tiab] OR “nutrition advice”[tiab] OR “nutritional advice”[tiab] OR counseling[mesh] OR ((“Patient education as topic”[Mesh] OR “Early intervention (education)”[Mesh]) AND (diet*[tiab] OR nutrition*[tiab])) OR “Dietary advices”[tiab] OR “nutritional advices”[tiab] OR “nutrition advices”[tiab] OR “Dietary interventions”[tiab] OR “Nutrition interventions”[tiab] OR “Nutritional interventions”[tiab])

#3 Clinical events

#3.1

(“quality of life”[tiab] OR Quality of Life[Mesh]) AND (Disease Progression[Mesh] OR Recurrence[Mesh] OR Prognosis[Mesh] OR Neoplastic Processes[Mesh] OR Neoplasm Recurrence, Local[Mesh] OR prognosis[tiab] OR progression[tiab] OR recurrence[tiab] OR relapse[tiab] OR remission[tiab]) AND (Neoplasms/drug therapy[Mesh] OR Neoplasms/radiotherapy[Mesh] OR Neoplasms/therapy[Mesh] OR therapy[Title/Abstract] OR chemotherapy[Title/Abstract] OR radiotherapy[Title/Abstract] OR treatment[Title/Abstract]) AND (adverse effects[Subheading] OR Neoplasms/complications[MH]) AND (Neoplasms, Second Primary[Mesh] OR Neoplasms, Multiple Primary[Mesh] OR (cancer*[tiab] AND (new[Title/Abstract] OR second*[Title/Abstract] OR subsequent[Title/Abstract] OR additional[Title/Abstract] OR metachronous[Title/Abstract] OR multiple[Title/Abstract] OR synchronous[Title/Abstract])) AND (Mortality[Mesh] OR Survival Analysis[Mesh] OR mortality[Subheading]) OR survival[tiab] OR mortality[tiab]) AND predictive factor[tiab] OR predictive factors[tiab] OR prognostic factor[tiab] OR prognostic factors[tiab]

#3.2

((((Disease Progression[Mesh] OR Recurrence[Mesh] OR Prognosis[Mesh] OR Neoplastic Processes[Mesh] OR Neoplasm Recurrence, Local[Mesh] OR prognosis[tiab] OR progression[tiab] OR recurrence[tiab] OR relapse[tiab] OR remission[tiab]))) OR ((Neoplasms, Second Primary[Mesh] OR Neoplasms, Multiple Primary[Mesh] OR (cancer*[tiab] AND (new[Title/Abstract] OR second*[Title/Abstract] OR subsequent[Title/Abstract] OR additional[Title/Abstract] OR metachronous[Title/Abstract] OR multiple[Title/Abstract] OR synchronous[Title/Abstract])))) OR ((Mortality[Mesh] OR Survival Analysis[Mesh] OR mortality[Subheading]) OR survival[tiab] OR mortality[tiab]))

#4 Type of studies

#4.1 Clinical trials + meta-analyses + pooled analyses

(“Clinical Trials as Topic”[Mesh:NoExp] OR “Clinical Trials, Phase I as Topic”[Mesh] OR “Clinical Trials, Phase II as Topic”[Mesh] OR “Clinical Trials, Phase III as Topic”[Mesh] OR “Clinical Trials, Phase IV as Topic”[Mesh] OR “Controlled Clinical Trials as Topic”[Mesh] OR “Clinical Trial”[Publication Type:NoExp] OR “Clinical Trial, Phase I”[Publication Type] OR “Clinical Trial, Phase II”[Publication Type] OR “Clinical Trial, Phase III”[Publication Type] OR “Clinical Trial, Phase IV”[Publication Type] OR “Controlled Clinical Trial”[Publication Type] OR “Randomized Controlled Trial”[Publication Type] OR Women’s Intervention Nutrition Study[TIAB] OR WHI[TIAB] OR Women’s Health Initiative[TIAB] OR WHEL[TIAB] OR Women’s Healthy Eating and Living Study[TIAB] OR DEDICa[TIAB] OR Diet, Exercise and Vitamin D in Breast Cancer Recurrence[TIAB] OR Choice trial[TIAB] OR Choosing Healthier Drinking Options In primary CarE[TIAB] OR “Meta-Analysis”[Publication Type] OR “Meta-Analysis as Topic”[Mesh] OR metaanalysis[title] OR “meta analysis”[title] OR “pooled analysis”[title])

#4.2 Cohort studies

(cohort[tiab] OR follow-up[tiab] OR longitudinal[tiab])

#5 Limits:

(French[la] OR English[la])

(“2010/08/01”[PDAT]: “2020/07/03”[PDAT])

hasabstract[text]

- Request A: #1 AND (#2.1 OR #2.2 OR #2.3 OR #2.4 OR #2.5) AND #3.1 AND #4.1 AND #5
- Request B: #1 AND (#2.1 OR #2.2 OR #2.3 OR #2.4 OR #2.5) AND #3.2 AND #4.2 AND #5

For the factor “weight”, filters were applied to obtain only the following cancer locations: endometrium, cervix, liver, pancreas, head and neck, pharynx, nasopharynx

- Request Nutritional advices: #1 AND #2.6 AND #3.1 AND #4.1 AND #5

## References

[1] Institut National du Cancer (2019). Les Cancers en France—L’essentiel des Faits et Chiffres.

[2] Institut National du Cancer (2016). Survie Nette Conditionnelle Chez les Personnes Atteintes de Cancer en France Métropolitaine.

[3] Demark-Wahnefried W., Aziz N.M., Rowland J.H., Pinto B.M. (2005). Riding the crest of the teachable moment: Promoting long-term health after the diagnosis of cancer. J. Clin. Oncol..

[4] Institut National du Cancer (2018). La vie Cinq ans Après un Diagnostic de Cancer.

[5] Institut National du Cancer (2015). Nutrition et Prévention Primaire des Cancers: Actualisation des Données, Collection État des Lieux et des Connaissances.

[6] Rock C.L., Doyle C., Demark-Wahnefried W., Meyerhardt J., Courneya K.S., Schwartz A.L., Bandera E.V., Hamilton K.K., Grant B., McCullough M. (2012). Nutrition and physical activity guidelines for cancer survivors. CA Cancer J. Clin..

[7] INCa (2017). Bénéfices de l’activité physique pendant et après cancer. Des Connaissances Scientifiques aux Repères Pratiques—Collection Etats des Lieux et des Connaissances.

[8] Arends J., Bachmann P., Baracos V., Barthelemy N., Bertz H., Bozzetti F., Fearon K., Hütterer E., Isenring E., Kaasa S. (2017). ESPEN guidelines on nutrition in cancer patients. Clin. Nutr..

[9] Chan D.S.M., Vieira A.R., Aune D., Bandera E.V., Greenwood D.C., McTiernan A., Rosenblatt D.N., Thune I., Vieira R., Norat T. (2014). Body mass index and survival in women with breast cancer—systematic literature review and meta-analysis of 82 follow-up studies. Ann. Oncol..

[10] Nechuta S., Chen W.Y., Cai H., Poole E.M., Kwan M.L., Flatt S.W., Patterson R.E., Pierce J.P., Caan B., Shu X.O. (2016). A pooled analysis of post-diagnosis lifestyle factors in association with late estrogen-receptor-positive breast cancer prognosis. Int. J. Cancer.

[11] Pajares B., Pollán M., Martín M., Mackey J.R., Lluch A., Gavila J., Vogel C., Ruiz-Borrego M., Calvo L., Pienkowski T. (2013). Obesity and survival in operable breast cancer patients treated with adjuvant anthracyclines and taxanes according to pathological subtypes: A pooled analysis. Breast Cancer Res. BCR.

[12] Fontanella C., Lederer B., Gade S., Vanoppen M., Blohmer J.U., Costa S.D., Denkert C., Eidtmann H., Gerber B., Hanusch C. (2015). Impact of body mass index on neoadjuvant treatment outcome: A pooled analysis of eight prospective neoadjuvant breast cancer trials. Breast Cancer Res. Treat..

[13] Druesne-Pecollo N., Touvier M., Barrandon E., Chan D.S.M., Norat T., Zelek L., Hercberg S., Latino-Martel P. (2012). Excess body weight and second primary cancer risk after breast cancer: A systematic review and meta-analysis of prospective studies. Breast Cancer Res. Treat..

[14] Wu S., Liu J., Wang X., Li M., Gan Y., Tang Y. (2014). Association of obesity and overweight with overall survival in colorectal cancer patients: A meta-analysis of 29 studies. Cancer Causes Control.

[15] Lee J., Meyerhardt J.A., Giovannucci E., Jeon J.Y. (2015). Association between Body Mass Index and Prognosis of Colorectal Cancer: A Meta-Analysis of Prospective Cohort Studies. PLoS ONE.

[16] Sinicrope F.A., Foster N.R., Yoon H.H., Smyrk T.C., Kim G.P., Allegra C.J., Yothers G., Nikcevich D.A., Sargent D.J. (2012). Association of Obesity with DNA Mismatch Repair Status and Clinical Outcome in Patients with Stage II or III Colon Carcinoma Participating in NCCTG and NSABP Adjuvant Chemotherapy Trials. J. Clin. Oncol..

[17] Sinicrope F.A., Ms N.R.F., Yothers G., Benson A., Seitz J.F., Labianca R., Goldberg R.M., Degramont A., O’Connell M.J., Sargent D. (2013). Body mass index at diagnosis and survival among colon cancer patients enrolled in clinical trials of adjuvant chemotherapy. Cancer.

[18] Zhang J., Chen Q., Li Z.-M., Xu X.-D., Song A.-F., Wang L.S. (2018). Association of body mass index with mortality and postoperative survival in renal cell cancer patients, a meta-analysis. Oncotarget.

[19] Hong L., Zhang H., Zhao Q., Han Y., Yang J., Brain L. (2013). Relation of excess body weight and survival in patients with esophageal adenocarcinoma: A meta-analysis. Dis. Esophagus.

[20] Wang J., Xu H., Zhou S., Wang D., Zhu L., Hou J., Tang J., Zhao J., Zhong S. (2018). Body mass index and mortality in lung cancer patients: A systematic review and meta-analysis. Eur. J. Clin. Nutr..

[21] Wu X.-S., Wu W.-G., Li M.-L., Yang J.-H., Ding Q.-C., Zhang L., Mu J.-S., Gu J., Dong P., Lu J.-H. (2013). Impact of being overweight on the surgical outcomes of patients with gastric cancer: A meta-analysis. World J. Gastroenterol..

[22] Patel J., Pereira J., Chen J., Liu J., Guba S., John W., Orlando M., Scagliotti G., Bonomi P. (2016). Relationship between efficacy outcomes and weight gain during treatment of advanced, non-squamous, non-small-cell lung cancer patients. Ann. Oncol..

[23] Renfro L.A., Loupakis F., Adams R., Seymour M.T., Heinemann V., Schmoll H.-J., Douillard J.-Y., Hurwitz H.I., Fuchs C.S., Diaz-Rubio E. (2016). Body Mass Index Is Prognostic in Metastatic Colorectal Cancer: Pooled Analysis of Patients From First-Line Clinical Trials in the ARCAD Database. J. Clin. Oncol..

[24] Aparicio T., Ducreux M., Faroux R., Barbier E., Manfredi S., Lecomte T., Etienne P.-L., Bedenne L., Bennouna J., Phelip J.-M. (2018). Overweight is associated to a better prognosis in metastatic colorectal cancer: A pooled analysis of FFCD trials. Eur. J. Cancer.

[25] Boshier P.R., Heneghan R., Markar S.R., Baracos V.E., Low D.E. (2018). Assessment of body composition and sarcopenia in patients with esophageal cancer: A systematic review and meta-analysis. Dis. Esophagus.

[26] Zhao B., Zhang J., Zhang J., Zou S., Luo R., Xu H., Huang B. (2018). The Impact of Preoperative Underweight Status on Postoperative Complication and Survival Outcome of Gastric Cancer Patients: A Systematic Review and Meta-analysis. Nutr. Cancer.

[27] Kamarajah S.K., Bundred J., Tan B.H.L. (2018). Body composition assessment and sarcopenia in patients with gastric cancer: A systematic review and meta-analysis. Gastric Cancer.

[28] Chang K.-V., Chen J.-D., Wu W.-T., Huang K.-C., Hsu C.-T., Han D.-S. (2017). Association between Loss of Skeletal Muscle Mass and Mortality and Tumor Recurrence in Hepatocellular Carcinoma: A Systematic Review and Meta-Analysis. Liver Cancer.

[29] Mintziras I., Miligkos M., Wächter S., Manoharan J., Maurer E., Bartsch D.K. (2018). Sarcopenia and sarcopenic obesity are significantly associated with poorer overall survival in patients with pancreatic cancer: Systematic review and meta-analysis. Int. J. Surg..

[30] Ren G., Cai W., Wang L., Huang J., Yi S., Lu L., Wang J. (2018). Impact of body mass index at different transplantation stages on postoperative outcomes in patients with hematological malignancies: A meta-analysis. Bone Marrow Transplant..

[31] Vrieling A., Kampman E., Knijnenburg N.C., Mulders P.F., Sedelaar J.M., Baracos V.E., Kiemeney L.A. (2018). Body Composition in Relation to Clinical Outcomes in Renal Cell Cancer: A Systematic Review and Meta-analysis. Eur. Urol. Focus.

[32] Shachar S.S., Williams G.R., Muss H.B., Nishijima T.F. (2016). Prognostic value of sarcopenia in adults with solid tumours: A meta-analysis and systematic review. Eur. J. Cancer.

[33] Clark L.H., Jackson A.L., Soo A.E., Orrey D.C., Gehrig P.A., Kim K.H. (2016). Extremes in body mass index affect overall survival in women with cervical cancer. Gynecol. Oncol..

[34] Kizer N.T., Thaker P.H., Gao F., Zighelboim I., Powell M.A., Rader J.S., Mutch D.G., Grigsby P.W. (2010). The effects of body mass index on complications and survival outcomes in patients with cervical carcinoma undergoing curative chemoradiation therapy. Cancer.

[35] Oei R.W., Ye L., Huang J., Kong F., Xu T., Shen C., Wang X., He X., Kong L., Hu C.-S. (2018). Prognostic value of nutritional markers in nasopharyngeal carcinoma patients receiving intensity-modulated radiotherapy: A propensity score matching study. OncoTargets Ther..

[36] Zeng Q., Shen L.-J., Guo X., Guo X.-M., Qian C.-N., Wu P.-H. (2016). Critical weight loss predicts poor prognosis in nasopharyngeal carcinoma. BMC Cancer.

[37] Pai P.-C., Chuang C.-C., Tseng C.-K., Tsang N.-M., Chang K.-P., Yen T.-C., Liao C.-T., Hong J.-H., Chang J.T.-C. (2012). Impact of Pretreatment Body Mass Index on Patients with Head-and-Neck Cancer Treated with Radiation. Int. J. Radiat. Oncol..

[38] Ravasco P., Monteiro-Grillo I., Camilo M. (2012). Individualized nutrition intervention is of major benefit to colorectal cancer patients: Long-term follow-up of a randomized controlled trial of nutritional therapy. Am. J. Clin. Nutr..

[39] Druesne-Pecollo N., Keita Y., Touvier M., Chan D.S., Norat T., Hercberg S., Latino-Martel P. (2014). Alcohol Drinking and Second Primary Cancer Risk in Patients with Upper Aerodigestive Tract Cancers: A Systematic Review and Meta-analysis of Observational Studies. Cancer Epidemiol. Biomark. Prev..

[40] Xing M.-Y., Xu S.-Z., Shen P. (2014). Effect of Low-fat Diet on Breast Cancer Survival: A Meta-analysis. Asian Pac. J. Cancer Prev..

[41] Chlebowski R.T., Aragaki A.K., Anderson G.L., Thomson C.A., Manson J.E., Simon M.S., Howard B.V., Rohan T.E., Snetselar L., Lane D. (2017). Low-Fat Dietary Pattern and Breast Cancer Mortality in the Women’s Health Initiative Randomized Controlled Trial. J. Clin. Oncol..

[42] Pettersson A., Kasperzyk J.L., Kenfield S.A., Richman E.L., Chan J.M., Willett W.C., Stampfer M.J., Mucci L.A., Giovannucci E.L. (2012). Milk and Dairy Consumption among Men with Prostate Cancer and Risk of Metastases and Prostate Cancer Death. Cancer Epidemiol. Biomark. Prev..

[43] Yang M., Kenfield S.A., Van Blarigan E.L., Wilson K.M., Batista J.L., Sesso H.D., Ma J., Stampfer M.J., Chavarro J.E. (2015). Dairy intake after prostate cancer diagnosis in relation to disease-specific and total mortality. Int. J. Cancer.

[44] Richman E.L., Kenfield S.A., Chavarro J.E., Stampfer M.J., Giovannucci E.L., Willett W.C., Chan J.M. (2013). Fat Intake After Diagnosis and Risk of Lethal Prostate Cancer and All-Cause Mortality. JAMA Intern. Med..

[45] Van Blarigan E.L., Kenfield S.A., Yang M., Sesso H.D., Ma J., Stampfer M.J., Chan J., Chavarro J.E. (2015). Fat intake after prostate cancer diagnosis and mortality in the Physicians’ Health Study. Cancer Causes Control.

[46] Chi F., Wu R., Zeng Y.-C., Xing R., Liu Y., Xu Z.-G. (2013). Post-diagnosis Soy Food Intake and Breast Cancer Survival: A Meta-analysis of Cohort Studies. Asian Pac. J. Cancer Prev..

[47] AFSSA/AFSSAPS (2005). Sécurité et Bénéfices des Phyto-Estrogènes Apportés par L’alimentation; Recommandations.

[48] Académie Nationale de Pharmacie (2018). Les Compléments Alimentaires Contenant des Plantes.

[49] World Cancer Research Fund/American Institute for Cancer Research (2014). Survivors of Breast Cancers. Continuous Update Project Expert Report: Diet, Nutrition, Physical Activity and Breast Cancer Survivors.

[50] Guercio B.J., Sato K., Niedzwiecki D., Ye X., Saltz L.B., Mayer R.J., Mowat R.B., Whittom R., Hantel A., Benson A. (2015). Coffee Intake, Recurrence, and Mortality in Stage III Colon Cancer: Results from CALGB 89803 (Alliance). J. Clin. Oncol..

[51] Hu Y., Ding M., Yuan C., Wu K., Smith-Warner S.A., Hu F.B., Chan A.T., Meyerhardt J.A., Ogino S., Fuchs C.S. (2018). Association between Coffee Intake after Diagnosis of Colorectal Cancer and Reduced Mortality. Gastroenterology.

[52] Harris H.R., Orsini N., Wolk A. (2014). Vitamin C and survival among women with breast cancer: A Meta-analysis. Eur. J. Cancer.

[53] Poole E.M., Shu X., Caan B.J., Flatt S.W., Holmes M.D., Lu W., Kwan M.L., Nechuta S.J., Pierce J.P., Chen W.Y. (2013). Postdiagnosis supplement use and breast cancer prognosis in the After Breast Cancer Pooling Project. Breast Cancer Res. Treat..

[54] Chen L., Chen Y., Wang X., Li H., Zhang H., Gong J., Shen S., Yin W., Hu H. (2015). Efficacy and safety of oral branched-chain amino acid supplementation in patients undergoing interventions for hepatocellular carcinoma: A meta-analysis. Nutr. J..

[55] Nojiri S., Fujiwara K., Shinkai N., Iio E., Joh T. (2016). Effects of branched-chain amino acid supplementation after radiofrequency ablation for hepatocellular carcinoma: A randomized trial. Nutrition.

[56] Asher G.N., Corbett A.H., Hawke R.L. (2017). Common Herbal Dietary Supplement-Drug Interactions. Am. Fam. Physician.

[57] Fasinu P.S., Rapp G.K. (2019). Herbal Interaction with Chemotherapeutic Drugs—A Focus on Clinically Significant Findings. Front. Oncol..

[58] Yetley E.A. (2007). Multivitamin and multimineral dietary supplements: Definitions, characterization, bioavailability, and drug interactions. Am. J. Clin. Nutr..

[59] Fai C.K., Chung L.P. (2012). Efficacy of Yun Zhi (Coriolus versicolor) on Survival in Cancer Patients: Systematic Review and Meta-Analysis. Recent Patents Inflamm. Allergy Drug Discov..

[60] Shi Q., Li W., Le Q.-Q., Chen W.-T., Ren J.-L., Li Q., Hou F.-G. (2016). Attenuated effects of Jianpi Qushi herbs on patients receiving FOLFOX4 after colorectal cancer surgery: A meta-analysis. Chin. J. Integr. Med..

[61] Zhang S., Shi L., Mao D., Peng W., Sheng C., Ding C., Lin F., Lei C., Zhang S. (2018). Use of Jianpi Jiedu Herbs in Patients with Advanced Colorectal Cancer: A Systematic Review and Meta-Analysis. Evid. Based Complement. Altern. Med..

